# The Role of FGF19 and MALRD1 in Enterohepatic Bile Acid Signaling

**DOI:** 10.3389/fendo.2021.799648

**Published:** 2022-01-18

**Authors:** Linda X. Wang, Mark R. Frey, Rohit Kohli

**Affiliations:** Division of Gastroenterology, Hepatology and Nutrition, Children’s Hospital Los Angeles, Keck School of Medicine, University of Southern California, Los Angeles, CA, United States

**Keywords:** MALRD1, FGF19, bile acid signaling, lipid metabolism, glucose metabolism

## Abstract

Bile acids are the catabolic end products of cholesterol metabolism that are best known for their role in the digestion of lipids. In the last two decades, extensive investigation has shown bile acids to be important signaling molecules in metabolic processes throughout the body. Bile acids are ligands that can bind to several receptors, including the nuclear receptor farnesoid X receptor (FXR) in ileal enterocytes. FXR activation induces the expression of fibroblast growth factor (FGF) 15/19, a hormone that can modulate bile acid levels, repress gluconeogenesis and lipogenesis, and promote glycogen synthesis. Recent studies have described a novel intestinal protein, MAM and LDL Receptor Class A Domain containing 1 (MALRD1) that positively affects FGF15/19 levels. This signaling pathway presents an exciting target for treating metabolic disease and bile acid-related disorders.

## Introduction

Bile acids are physiological detergent molecules synthesized from cholesterol that were once thought to primarily function in the intestinal tract to solubilize and facilitate absorption of fats, steroids, and fat-soluble vitamins. However, more recent research has revealed their broader role as signaling molecules that activate nuclear receptors and G protein-coupled receptors to modulate a variety of metabolic processes such as glucose homeostasis, lipid metabolism, immune cell function, and cell growth and proliferation (1). Their role in these processes requires a tightly regulated cyclical process of bile acid synthesis in the liver, transport, and reabsorption from the ileum to maintain precise levels in circulation. Any alteration in this homeostasis affects hepatic metabolic processes, potentially resulting in inflammation and development of diseases such as cholestatic liver diseases, dyslipidemia, diabetes, and even cancer (1, 2). Numerous studies have demonstrated the importance of the hormone, fibroblast growth factor 19 (FGF19), in the maintenance of this homeostasis. Most recently, a newly identified intestinal protein, MALRD1, has been shown to modulate levels of FGF19 (3, 4). This review will provide an overview of bile acid metabolism, enterohepatic bile acid signaling, metabolic effects of FGF19, and MALRD1.

## Bile Acid Metabolism

### Bile Acid Synthesis

**Figure 1:**
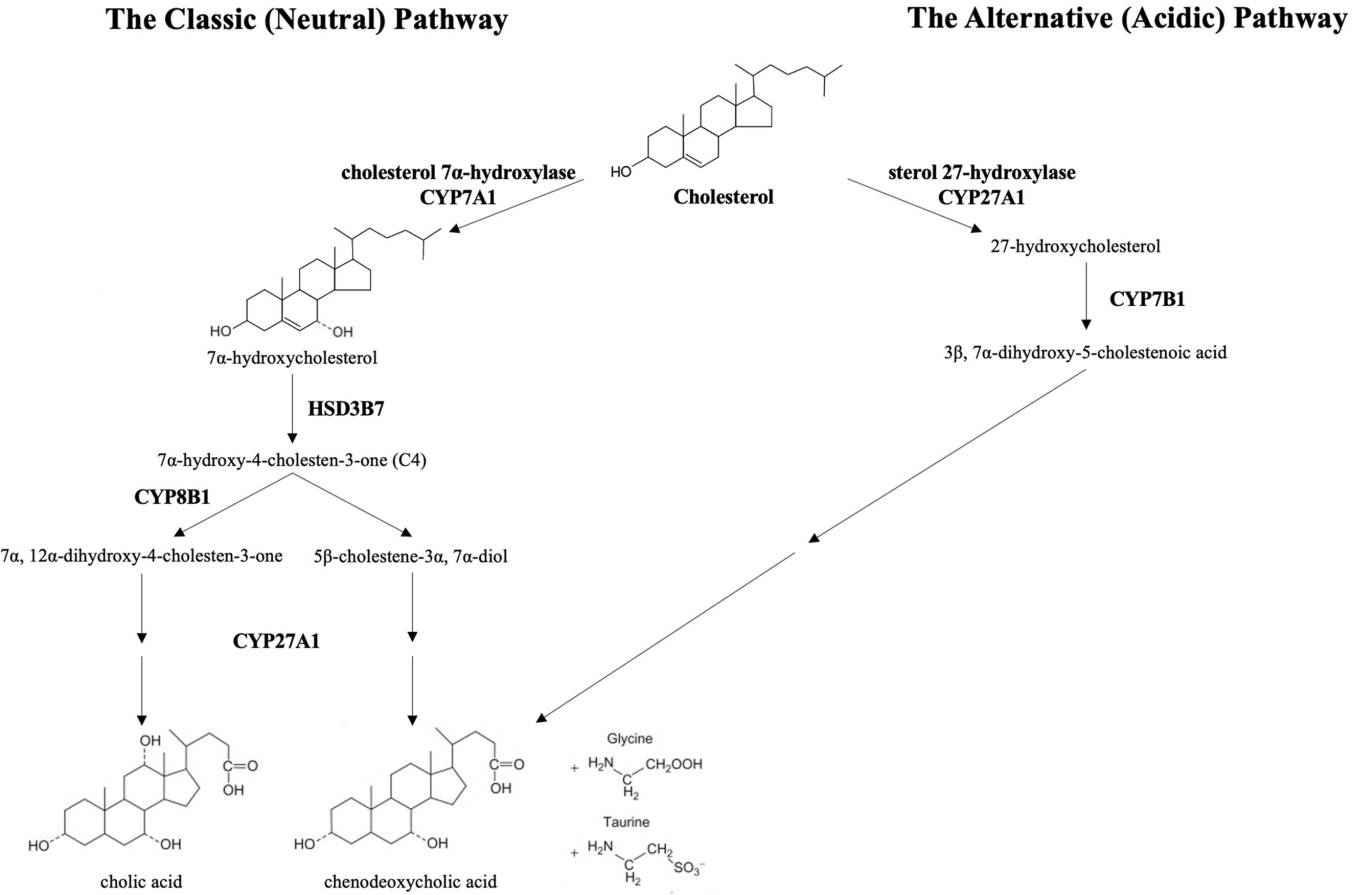
The classic and alternative bile synthetic pathways. CYP7A1, cholesterol 7α-hydroxylase, is the rate limiting enzyme in the classic (neutral) pathway. HSD3B7, 3 beta-hydroxysteroid dehydrogenase type 7, creates the common precursor, 7α-hydroxy-4-cholesten-3-one (C4), for both primary bile acids. CYP8B1, sterol 12-alpha-hydroxylase, initiates the pathway toward the formation of cholic acid. In the acidic pathway, CYP27A1, sterol 27-hydroxylase, catalyzes the first step toward the formation of chenodeoxycholic acid. The primary bile acids are conjugated to the amino acids, glycine and taurine, prior to excretion into the biliary system.

Bile acids are synthesized from cholesterol through a complex multi-enzyme series of reactions in hepatocytes. The adult human bile acid pool consists of approximately 40% cholic acid (CA), 40% chenodeoxycholic acid (CDCA), 20% secondary bile acid deoxycholic acid (DCA), and trace amounts of lithocholic acid (LCA) (5). There are two main pathways responsible for bile acid synthesis **(** **Figure 1** **).** In the neutral or classic bile acid pathway, the rate-limiting cytochrome P450 enzyme cholesterol 7α-hydroxycholesterol (CYP7A1) initiates the conversion of cholesterol to the primary bile acids, CA and CDCA. The intermediate product 7α-hydroxy-4-cholestene-3-one (C4) is the common precursor for these two bile acids. The microsomal CYP enzyme, sterol 12α-hydroxylase (CYP8B1), is required for synthesis of CA.

In the acidic or alternative pathway, cholesterol 27-hydroxylase (CYP27A1), a mitochondrial P450 enzyme, catalyzes the first reaction that leads to the final production of CDCA (6). The acidic pathway contributes to less than 10% of the total bile acid production in humans (7). Of note, the acidic pathway has been found to be more important in those with liver disease and in neonates (8, 9).

### Bile Acid Conjugation

The primary bile acids, CA and CDCA, are conjugated with the amino acids glycine and taurine in a 3:1 ratio, depending on the availability of dietary taurine and animal species (e.g., in mice most bile acids are taurine-conjugated) (10, 11). Conjugation serves to increase the solubility of the bile acids and enables their transport *via* bile acid transporters on hepatocytes into the bile canalicular system and subsequently into the gallbladder. It also limits their passive reabsorption as they pass down the biliary system. After a meal, the secretion of cholecystokinin induces gallbladder contraction, which releases its contents into the gastrointestinal tract (5). Greater than 70% of the stored bile is expelled into the proximal small intestine.

### Enterohepatic Circulation

**Figure 2:**
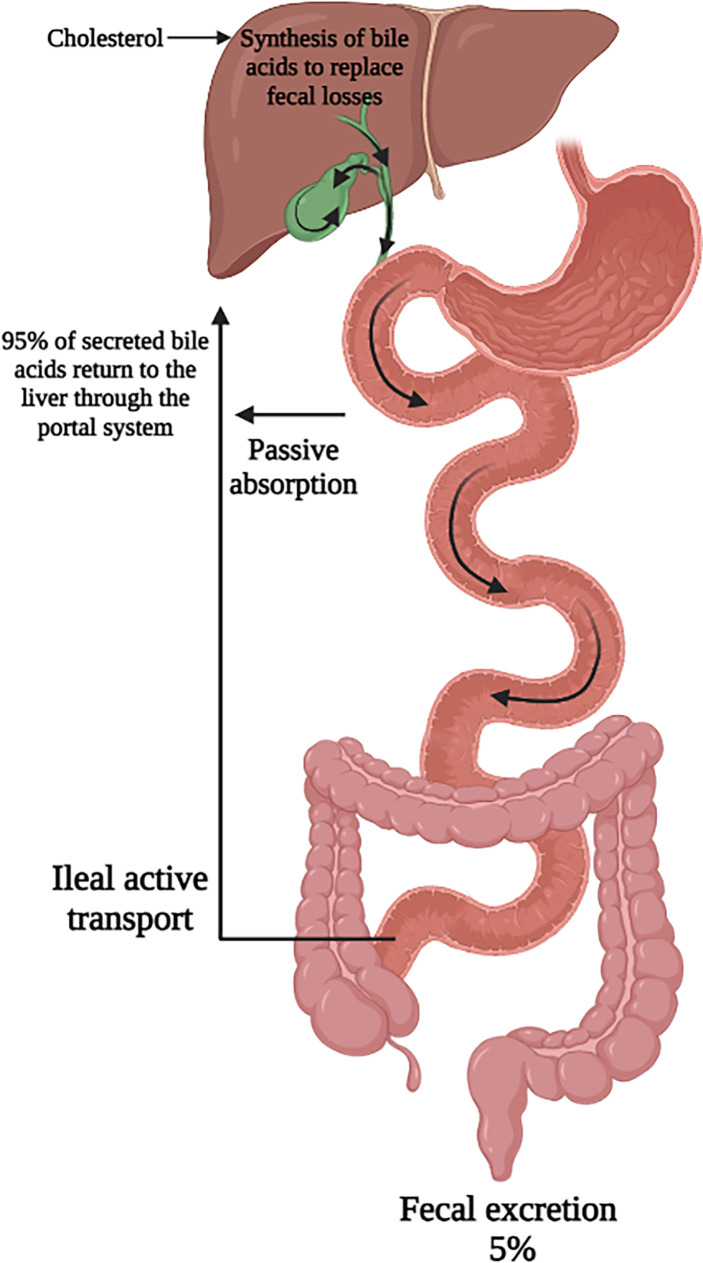
Enterohepatic circulation of bile acids. Secreted bile acids are passively absorbed (minimally) in the proximal intestine and actively transported (majority) in the ileum. This allows for recovery of 95% of secreted bile acids back to the liver. Figure created on biorender.com.

A minority of secreted bile acids are passively reabsorbed from the proximal small intestine. The majority of bile acids secreted into the intestines each cycle are reabsorbed in the ileum by active transport back into the portal system and circulated back to the liver. Transporters on the apical membrane of ileal enterocytes and on the sinusoidal membrane of the hepatocyte are highly efficient in this process, with recovery of about 95% of luminal bile acids (1, 11, 12). The 5% lost in feces are replenished by *de novo* hepatic bile acid synthesis (13). This synthesis of bile acids is self-regulated by negative feedback by bile acids returning to the liver (14). This bile acid pool of approximately 4 to 5 g is recycled 6 to 10 times per day through the coordinated action of several bile acid transporters, the expression of which are controlled at the transcriptional level **(** **Figure 2** **)**. The flow of bile also facilitates hepatobiliary secretion of metabolites and xenobiotics (15).

### Bile Acid Transformation

The small percentage of primary bile acids that reach the colon undergo significant structural modifications by intestinal bacteria, leading to the formation of secondary bile acids, deoxycholic acid (DCA) and lithocholic acid (LCA), derived from CA and CDCA respectively. LCA is highly hepatotoxic and is mostly excreted into feces, whereas DCA circulates with the primary bile acids (16).

Bacterial deconjugation creates unconjugated mono- or dihydroxy bile acids, which can be passively absorbed through colonic epithelium and recycled back to the liver. The enzymes that catalyze these transformations are found in bacterial organisms such as *Bacteroidetes, Clostridium* species, *Bifidobacteriaceae*, and *Enterococcus* (7, 16). Gut bacteria are thought to benefit from bile metabolism through acquisition of glycine and taurine, which can be used as an energy source in metabolism (17, 18). Alterations in intestinal microbiota can therefore have effects on bile acid pool size and composition, relevant to multiple chronic disease states (19–21). The same is true in reverse as changes in intra-luminal bile acid pool can impact the composition of the gut microbiome.

## The Role of Bile Acids as Metabolic Regulators

Bile acids are now well recognized as ligands for multiple nuclear receptors, including the farnesoid X receptor (FXR), the vitamin D receptor (VDR), the pregnane X receptor (PXR), constitutive androstane receptor (CAR), and the G protein coupled receptor Takeda G protein receptor 5 (TGR5) (1). Activation of these receptors leads to regulation of genes integral to metabolic processes throughout the body including in the brain, intestine, liver, brown adipose tissue, and macrophages **(** **Table 1** **).** FXR has been implicated in the regulation of enterohepatic circulation, glucose, lipid, and energy metabolism, and tumorigenesis through its downstream effector, FGF 15/19 (mouse and human orthologues).

**Table 1:** Bile acid receptors and physiological functions.

Receptor	Representative Ligands	Cytogenetic location	Tissue/Cell Expression	Functions
FXR (NR1H4)	CDCA>DCA>LCA>CA; INT-747, GW4064, fexaramine, PX-102	12q23.1	Liver, intestine, kidney, adrenal gland	Bile acid metabolism (22–28) Glucose metabolism (29) Lipid metabolism (29) Liver regeneration (30) Anti-inflammatory (31)
VDR (NR1I1)	LCA; vitamin D; LY2108491	12q13.11	Liver, intestine, gallbladder, bone, kidney, parathyroid, skin, bone marrow	Bile acid synthesis (32) Xenobiotic detoxification (33) Calcium homeostasis (34) Antimicrobial defense (35)
PXR (NR1I2)	LCA, DCA, CA; progesterone; rifampicin	3q13.33	Liver, intestine, immune cells	Bile acid synthesis (36) Glucose metabolism (37) Lipid metabolism (37, 38) Drug metabolism (37) Anti-inflammatory (37, 39)
CAR (NR1I3)	CA, 6-keto-LCA, 12-keto LCA; phenobarbital	1q23.3	Liver, intestine, kidney	Xenobiotic detoxification (40, 41) Glucose metabolism (42, 43) Lipid metabolism (43)
TGR5	LCA>DCA>CDCA>CA; INT-767; oleanolic acid	2q35	Liver, intestine, gallbladder, muscle, brown adipose, brain	Glucose homeostasis (44) Intestinal motility (45) Gallbladder relaxation (46) Energy metabolism (47) Anti-inflammatory (48)

### Regulation of Enterohepatic Circulation

**Figure 3:**
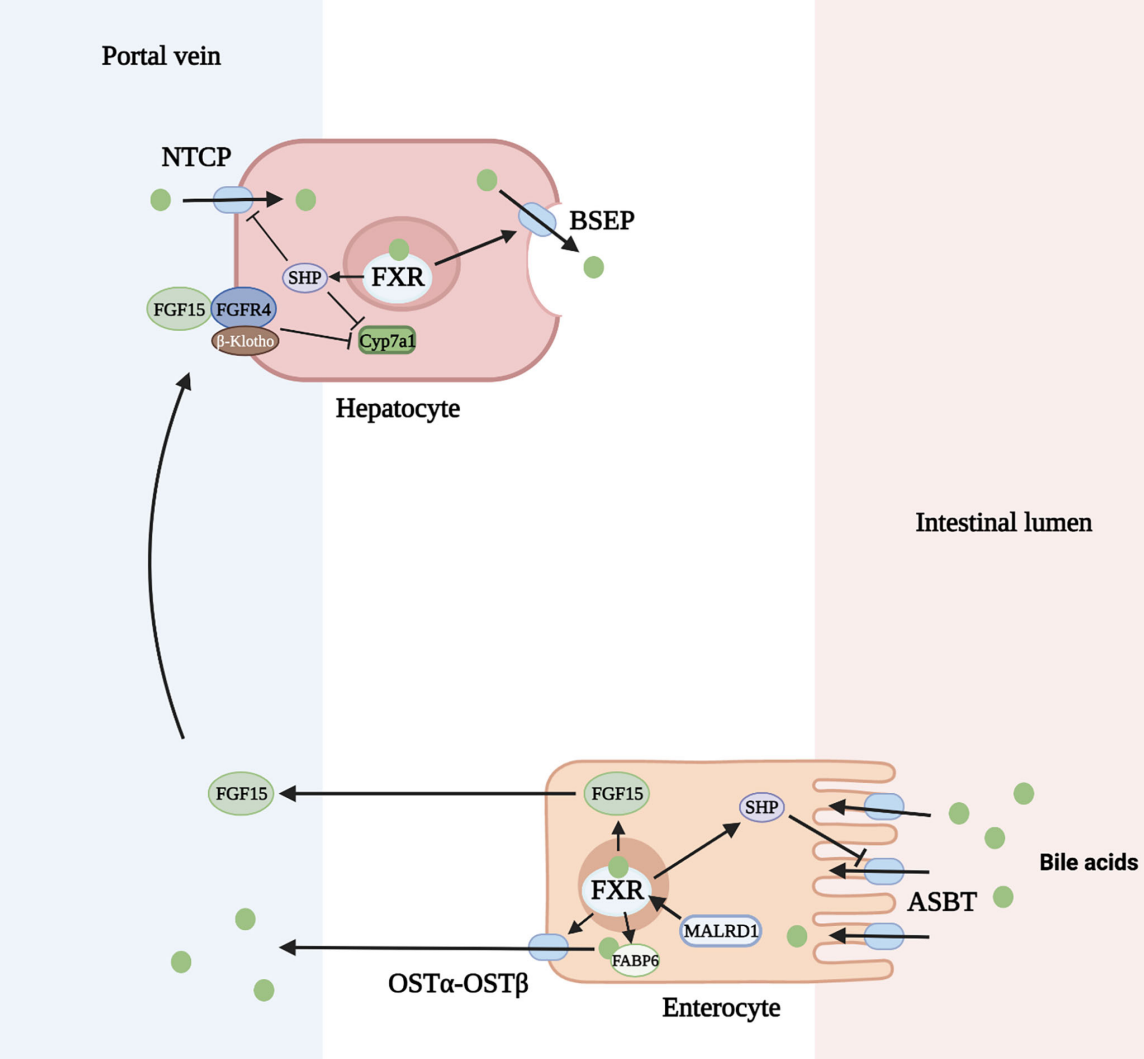
Regulation of bile acid homeostasis by FXR (farnesoid X receptor) and FGF15 (fibroblast growth factor 15). ASBT, apical sodium dependent bile acid transporter; BSEP, bile salt export pump; FGFR4, FGF receptor 4; FABP6, fatty acid binding protein 6; NTCP, Na+-taurocholate cotransporting polypeptide; OSTα-OSTβ, organic solute transporter α and β; SHP, short heterodimeric partner. Figure created on biorender.com.

The enterohepatic circulation of bile acids serves to control bile acid synthesis through a negative feedback mechanism by activation of FXR in the intestine and liver. In the ileum, bile acids are transported across the apical membrane via the apical sodium dependent bile acid transporter (ASBT). They bind to FXR and induce expression of the gene encoding FGF 15/19. FGF 15/19 is then secreted through the circulation to the liver where it binds to the FGF receptor (FGFR) 4 and its co-receptor, β-Klotho, on the hepatocyte cell membrane. Through subsequent activation of both extracellular signal-regulated kinase (ERK) and Jun N-terminal kinase (JNK) pathways, *CYP7A1* transcription is downregulated. In the liver, activation of FXR induces expression of *SHP (*encodes short heterodimeric partner, SHP) which inhibits *CYP8B1* transcription and to a lesser degree *CYP7A1* (22) **(** **Figure 3** **)**. Rats fed with bile acids showed strongly reduced activity of CYP7A1 and bile acid synthesis, whereas interruption of the enterohepatic circulation with use of bile acid binding resins (e.g. cholestyramine) increased the activity of CYP7A1 (49). A study in mice deficient in ileal apical sodium-dependent bile acid transporter (ASBT, *SLC10A2*) showed reduced intestine *Fgf15* expression, higher hepatic *Cyp7A1* expression and resistance to atherosclerosis development (50). Additionally, mice lacking the intestinal basolateral bile acid transporter, organic solute transporter α and β (OSTα-OSTβ, *SLC51A-SLC51B*), exhibited reduced bile acid pool size with a decrease in hepatic bile acid synthesis resulting from intracellular bile acid retention and increased *Fgf15* expression (51).

In addition to inhibition of bile acid synthesis, FXR activation also regulates bile acid transport. FXR inhibits hepatic bile acid uptake and ileal bile acid uptake through decreased production of the sodium-dependent transporter, Na^+^-taurocholate cotransporting polypeptide (NTCP, *SLC10A1*), and ASBT, respectively. This is mediated through induction of SHP which inhibits the retinoic acid and retinoid X receptor (RAR/RXR) heterodimer on the gene promoter (23, 24). FXR^+/+^ mice fed a 1% cholic acid diet show a marked reduction of NTCP RNA levels. FXR^-/-^ mice fed the same diet show no change in NTCP or bile acid import (25). In contrast, FXR activation upregulates the bile salt export pump (BSEP, *ABCB11*) on the apical membrane of hepatocytes and OSTα-OSTβ transporter on the apical membrane of enterocytes. FXR forms a heterodimer with retinoid X receptor (RXR) which binds to an inverted repeat (IR)-1 element on the gene promoter for BSEP or OSTα-OSTβ to induce the transporter production in a positive feed forward manner (26). This mechanism has been demonstrated by the induction of *ABCB11* mRNA and protein in mice fed a large dose of bile acid. FXR-deficient mice have low levels of BSEP that do not change after being fed a bile-acid enriched diet (25). Similarly, elevated *SLC51A-SLC51B* mRNA levels were seen after wild-type mice were administered a synthetic FXR agonist, but this response was decreased in FXR-null mice (27). Lastly, the production of cytosolic intestinal bile acid binding protein (fatty acid binding protein 6, FABP6) is upregulated by bile acids through activation of nuclear FXR allowing increased transport of bile acids through the enterocyte (28).

It is evident that FXR and FGF 15/19 have essential roles in the enterohepatic circulation of bile acids. Dysregulation of FXR target genes not only impairs enterohepatic circulation, but also results in cholestatic disease.

### Glucose Metabolism

**Figure 4:**
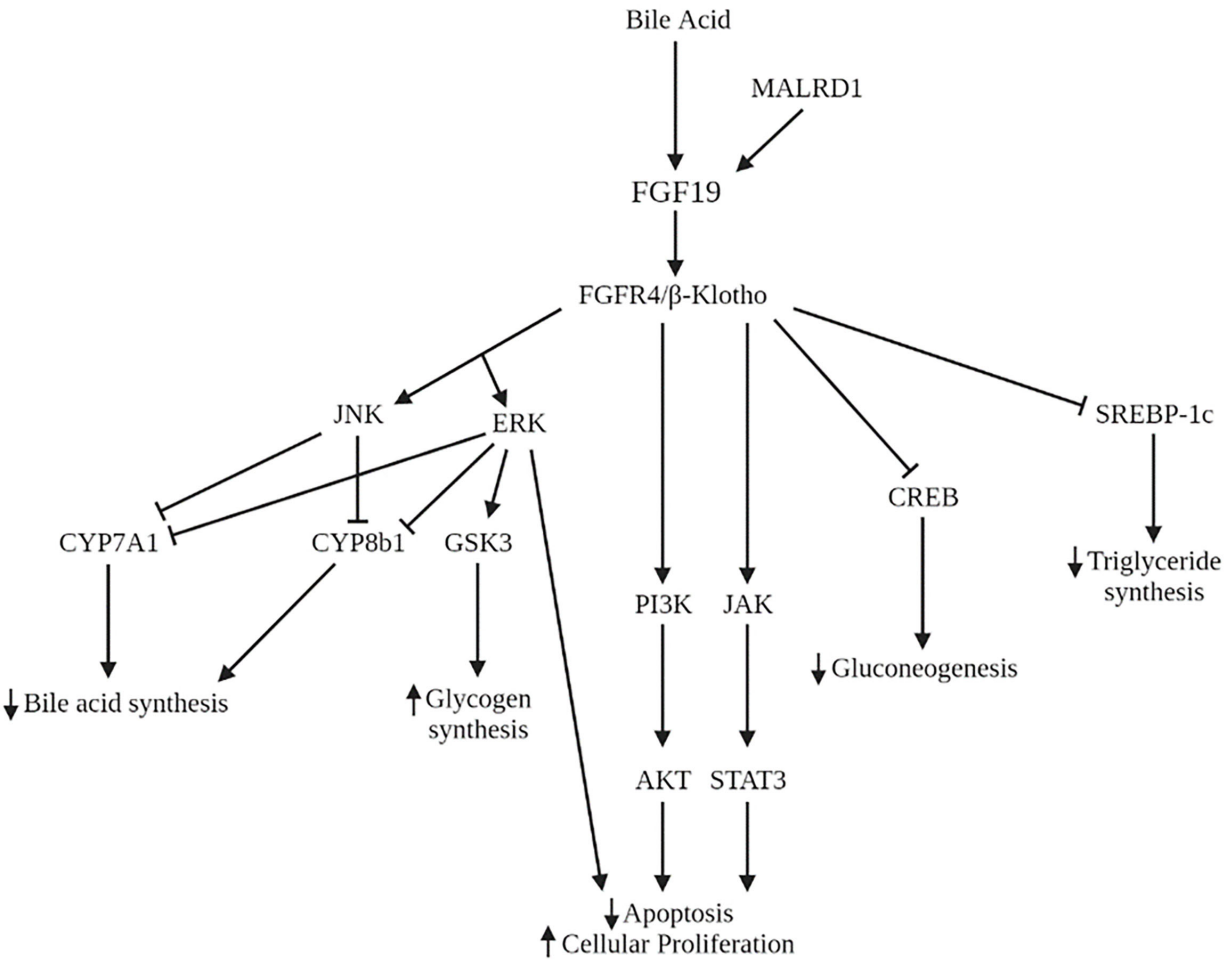
Signaling pathways of FGF19 and MALRD1. AKT, serine/threonine protein kinase B**;** CREB, cMAP response element-binding protein; ERK, extracellular signal-regulated kinase; GSK3, glycogen synthase kinase; JAK, Janus kinase; JNK, Jun N-terminal kinase; PI3K, phosphoinositide 3-kinase; STAT3, signal transducer and activator of transcription 3; SREBP-1c, sterol regulatory element binding protein-1c.

Many studies have implicated the FXR-FGF19 signaling axis in regulation of hepatic glucose metabolism. FGF15/19 acts as a post-prandial hormone that enhances glycogen synthesis and inhibits gluconeogenesis independent of insulin. In humans, serum FGF19 concentrations peak 2 to 3 hours following a meal, with a half-life of 30 minutes (52, 53). Glycogen synthesis is mediated through activation of ERK pathway with increased phosphorylation of glycogen synthase kinase (GSK) 3α and GSK3β leading to decreased inhibition of glycogen synthase. FGF15 deficient mice have impaired glucose uptake and decreased hepatic glycogen compared to wild-type mice. Administration of FGF19 reverses these effects. Inhibition of gluconeogenesis occurs through inactivation of cMAP response element-binding protein (CREB) **(** **Figure 4** **)**. Both processes were shown to occur independently of insulin (54). However, insulin does increase hepatic FGFR4, indicating a priming of FGF19 action (55).

Previous research has shown that FGF19 administered to cerebral ventricles results in decreased food intake, lower glucose levels and improved insulin sensitivity in mice fed a high fat diet (56, 57) indicating a potential link between diabetes and FGF19. However, the data in humans has been inconsistent (58–60). The most suggestive evidence was noted in diabetic patients who have remission of diabetes after Roux-en-Y gastric bypass surgery. Pre-operatively, those with diabetes had lower FGF19 and bile acid levels than nondiabetic patients. Post-operatively, levels of FGF19 and bile acids rose higher in diabetic patients than nondiabetic patients (61).

### Lipid Metabolism

In addition to its role in carbohydrate storage, FGF19 also has long-term effects on hepatic lipid metabolism. FGF19 inhibits the synthesis of the transcription factor sterol regulatory element binding protein-1c (SREBP-1c), which activates the transcription of genes necessary for fatty acid synthesis. In addition, FGF19 increases expression of *SHP* which also acts to inhibit lipogenic enzyme synthesis (62) **(** **Figure 4** **)**. Administration of FGF19 to FXR-null mice prevented hepatic fat deposition and decreased liver enzyme levels (63). Clinically, pediatric patients with non-alcoholic fatty liver disease (NAFLD) have demonstrated decreased FGF19 levels (64, 65). FGF19 levels were shown to be inversely associated with development of steatohepatitis and fibrosis (64). Lastly, adults with NAFLD and insulin resistance show an impaired response to FGF19 compared to those with NAFLD and without insulin resistance (66). This impairment may further contribute to the dysregulation of lipid homeostasis in NAFLD.

Given the findings of these studies, there are emerging medical therapies for treatment of nonalcoholic steatohepatitis (NASH) that include agonists of FXR and FGF19 analogs. Of those in clinical trials, obeticholic acid (OCA), a FXR agonist, has been shown to improve fibrosis and prevent progression of fibrotic disease (67). An engineered FGF19 analog, NGM282, produced a significant reduction in liver fat content (>30%) in patients with NASH. Liver fat content normalized in up to 39% of patients (68).

### Energy Metabolism

By regulating both glucose and lipid metabolism, FGF19 plays a central role in energy balance. Several studies have observed that plasma FGF19 levels are significantly lower in obese patients compared to nonobese controls (69–71). A study of transgenic mice overexpressing FGF19 demonstrated increased energy expenditure with reduction in fat mass (72). When fed a high-fat diet, the transgenic mice did not become obese or diabetic. This is thought to be due to higher oxygen consumption, increased insulin sensitivity, and increase in brown adipose tissue activity. The same group later reported that administration of FGF19 to obese mice resulted in increased metabolic rate, decreased respiratory quotient, and prevention or reversal of diabetes (73).

These metabolic observations suggest that FGF19 may be a potential candidate for treatment of obesity. Modified forms of FGF19 have been created that have been effective in reducing body weight, plasma glucose and triglyceride levels in diet induced obesity (74). These variants were successful in uncoupling the metabolic effects of FGF19 from its effects on hepatocyte proliferation.

### Hepatocyte Growth and Proliferation

The FGF15/19-FGFR4 signaling pathway has been implicated in the initiation and progression of several cancers including lung, breast, colorectal, and hepatocellular carcinoma (HCC). The initial evidence of FGF19’s role in hepatocyte growth came from observations in FGF19 transgenic mice who developed hepatocellular carcinomas by 12 months of age. Administration of FGF19 led to increases in hepatocellular proliferation which preceded tumor development (75). Tumor formation in these mice was subsequently prevented by monoclonal antibodies that selectively blocked the interaction of FGF19 with FGFR4 (76) or by deficiency in FGFR4 (77). In humans, FGF19 and FGFR4 are both overexpressed in HCC compared to noncancerous liver tissue (76). FGF19 is an independent negative prognostic factor for survival (78). The mechanism underlying this process is the dysregulation of RAS-RAF-ERK1/12MAPK, PI3K-AKT, and STAT3 pathways leading to decreased apoptosis and increased cell growth and proliferation (79–81) (**Figure 4**). Furthermore, FGF19 facilitates resistance to apoptosis (82) and promotes metastasis (83) through GSK3β activation. Therapies aimed at inhibiting FGF19 and FGFR4 are in various phases of development, including clinical trials for several malignancies including HCC (84). Side effects of these therapies often include increased bile acid synthesis, change in bile acid transporter expression, and enhanced enterohepatic circulation.

## MALRD1, a Regulator of Bile Acid Signaling

The many roles of FGF15/19, as detailed above, in metabolism has spurred interest in potential regulators of its activity. In the last decade, one intestinal protein, MALRD1, has been identified that influences the production of FGF15/19 in enterocytes.

The locus was initially identified after an observation that a mouse sub-strain, C57BL/6ByJ (B6By), was resistant to diet-induced hypercholesterolemia and atherosclerosis seen in another closely related common strain, C57BL/6J (B6J) (85). There were however no differences noted in food consumption and cholesterol absorption. The B6By mice showed increased bile acid excretion and an enlarged bile acid pool with elevated expression of several genes involved in bile acid synthesis and transport, including *Cyp7a1* (86). It was established that serum bile acid levels co-segregate with cholesterol levels, indicating that the same locus was responsible for both phenotypes. Through genetic mapping and sequencing, the gene was identified on mouse chromosome 2 and named *Diet1*.

*Diet1* encodes a 236 kDa protein that contains nine copies of the MAM (meprin-A5-tyrosine phosphatase μ) domain which are interspersed with nine copies of the low-density lipoprotein receptor (LDLR) class A domain. The official gene name was since been changed to MAM and LDL Receptor Class A Domain containing 1 (*MALRD1* human, *Malrd1* mice). The amino acid sequence of MALRD1 is highly conserved between mouse and human at 70% identity, as well as other species including zebrafish, chicken, frog, and rat. MALRD1 is expressed mainly in the small intestine and in lower levels at the kidney cortex. Within the intestine, MALRD1 expression was localized to the epithelial cell layer (87).

### The Effects of MALRD1 on FGF 15/19

In the seminal paper on MALRD1, deficiency of this protein was shown to reduce ileal *Fgf15* mRNA and protein levels. B6By mice with rescued *Malrd1* expression had increased *Fgf15* mRNA and protein secretion by approximately 3-fold and reduced *Cyp7a1* mRNA levels (87). A similar effect in FGF15 protein secretion was seen with overexpression and knockdown of *Malrd1* in cultured intestinal cells. In contrast, *Fgf15* mRNA levels were minimally affected by either overexpression or knockdown of *Malrd1*. MALRD1 was shown to interact and co-localize with FGF19 within an intracellular compartment (87). Taken together, these data suggest that MALRD1 influences FGF15/19 levels at both the mRNA and post-transcriptional levels.

### Association With Disease

A recent study of Malrd1 deficient mice found that these mice not only had elevated bile acids and reduced FGF15 levels, but also reduced gastrointestinal transit and increased intestinal luminal water content (88). This is similar to the phenotype seen in patients with bile acid diarrhea (BAD), in which increased luminal bile acid levels induce water secretion and accelerate colonic transit. Thus far, there has been no reported association of *MALRD1* with clinical disease though many *MALRD1* variants have been found. One variant is noted to be more common in individuals with BAD and is associated with decreased levels of FGF19 secretion (3, 88).

A genetic analysis of patients with hepatocellular carcinoma found *MALRD1 (DIET1)* to be a co-expressing protein coding gene (PCG) for a long non-coding RNA sequence associated with hepatocellular carcinoma. Although the study did not show any diagnostic or prognostic value to *MALRD1* expression, it is interesting to note this association due to the role of FGF19 in hepatocellular growth and proliferation (89). Further studies will need to be undertaken to elucidate the relationship between *MALRD1* and the pathogenesis of this disease.

The extensive role of FGF19 in metabolic processes of lipid metabolism and glucose metabolism provides an intriguing potential of MALRD1 in the regulation of these processes as well. Studies are currently in place to explore the influence of MALRD1 in progression to NAFLD. The absence of MALRD1 can be theorized to result in increased hepatic steatosis and fibrosis due to its effects on the reduction of FGF19 levels.

A recent study examining the genetic risk factors for development of diabetic retinopathy revealed a single nucleotide polymorphism (SNP), rs12267418, located within the *MALRD1* gene to be associated with severe disease in Caucasians (90). Another study in a Chinese diabetic population also found 2 SNPs within the *MALRD1* gene, although an association was not found. This intriguing finding raises the question of whether ethnic differences or epigenetic effects may be responsible for the differing results (91).

Additionally, the role of MALRD1 in intestinal cellular proliferation and growth is being examined. Previously, administration of CDCA has been shown to induce FGF19, GLP-1, and GLP-2 levels along with increased intestinal mucosal growth *via* the FXR and TGR5 pathways. Further studies are being undertaken to elucidate the potential effects of MALRD1 on the TGR5 pathway.

## Conclusion

Research in the last few decades has revealed an important role for bile acids in the regulation of metabolic processes in the body. Maintenance of bile acid homeostasis is critical for prevention of metabolic disorders ranging from cholestasis to diabetes. The identification of components of bile acid signaling pathways has led to a deeper understanding of the regulatory mechanisms involved and provides a molecular basis for developing therapies for treatment of metabolic diseases. The newly discovered intestinal protein, MALRD1, offers another potential target for future research.

## Author Contributions

All authors contributed to the conception of the review framework. LW wrote and edited the manuscript. RK and MF provided guidance and edited the manuscript. All authors contributed to the article and approved the submitted version.

## Funding

This work was supported by North American Society for Pediatric Gastroenterology, Hepatology & Nutrition (NASPGHAN) Foundation/Nestlé Nutrition Research Young Investigator Development Grant.

## Conflict of Interest

The authors declare that the research was conducted in the absence of any commercial or financial relationships that could be construed as a potential conflict of interest.

## Publisher’s Note

All claims expressed in this article are solely those of the authors and do not necessarily represent those of their affiliated organizations, or those of the publisher, the editors and the reviewers. Any product that may be evaluated in this article, or claim that may be made by its manufacturer, is not guaranteed or endorsed by the publisher.
